# Sex Differences in the Physiological Network of Healthy Young Subjects

**DOI:** 10.3389/fphys.2021.678507

**Published:** 2021-05-11

**Authors:** Antonio Barajas-Martínez, Elizabeth Ibarra-Coronado, Ruben Fossion, Juan Claudio Toledo-Roy, Vania Martínez-Garcés, Juan Antonio López-Rivera, Geraldine Tello-Santoyo, Rusland D. Lavin, José Luis Gómez, Christopher R. Stephens, Carlos A. Aguilar-Salinas, Bruno Estañol, Nimbe Torres, Armando R. Tovar, Osbaldo Resendis-Antonio, Marcia Hiriart, Alejandro Frank, Ana Leonor Rivera

**Affiliations:** ^1^Doctorado en Ciencias Biomédicas, Universidad Nacional Autónoma de México, Mexico City, Mexico; ^2^Centro de Ciencias de la Complejidad, Universidad Nacional Autónoma de México, Mexico City, Mexico; ^3^Facultad de Medicina, Universidad Nacional Autónoma de México, Mexico City, Mexico; ^4^Instituto de Ciencias Nucleares, Universidad Nacional Autónoma de México, Mexico City, Mexico; ^5^Plan de Estudios Combinados en Medicina (PECEM-MD/PhD), Facultad de Medicina, Universidad Nacional Autónoma de México, Mexico City, Mexico; ^6^Facultad de Ciencias, Universidad Nacional Autónoma de México, Mexico City, Mexico; ^7^Instituto Nacional de Ciencias Médicas y Nutrición “Salvador Zubirán”, Mexico City, Mexico; ^8^Instituto Nacional de Medicina Genómica, Coordinación de la Investigación Científica-Red de Apoyo a la Investigación, UNAM, Mexico City, Mexico; ^9^Instituto de Fisiología Celular, Mexico City, Mexico; ^10^El Colegio Nacional, Mexico City, Mexico

**Keywords:** physiological network, sex differences, sexual dimorphism, heart rate variability, blood test, anthropometric measures, health

## Abstract

Within human physiology, systemic interactions couple physiological variables to maintain homeostasis. These interactions change according to health status and are modified by factors such as age and sex. For several physiological processes, sex-based distinctions in normal physiology are present and defined in isolation. However, new methodologies are indispensable to analyze system-wide properties and interactions with the objective of exploring differences between sexes. Here we propose a new method to construct complex inferential networks from a normalization using the clinical criteria for health of physiological variables, and the correlations between anthropometric and blood tests biomarkers of 198 healthy young participants (117 women, 81 men, from 18 to 27 years old). Physiological networks of men have less correlations, displayed higher modularity, higher small-world index, but were more vulnerable to directed attacks, whereas networks of women were more resilient. The networks of both men and women displayed sex-specific connections that are consistent with the literature. Additionally, we carried out a time-series study on heart rate variability (HRV) using Physionet’s Fantasia database. Autocorrelation of HRV, variance, and Poincare’s plots, as a measure of variability, are statistically significant higher in young men and statistically significant different from young women. These differences are attenuated in older men and women, that have similar HRV distributions. The network approach revealed differences in the association of variables related to glucose homeostasis, nitrogen balance, kidney function, and fat depots. The clusters of physiological variables and their roles within the network remained similar regardless of sex. Both methodologies show a higher number of associations between variables in the physiological system of women, implying redundant mechanisms of control and simultaneously showing that these systems display less variability in time than those of men, constituting a more resilient system.

## Introduction

The integration of the physiological systems that conform the human body and its operation can be considered an open structure with characteristics of a complex system that are the result of the large number of system components that comprise it, and the coupling and interactions between them (Bashan et al., 2012). The non-linear interaction of these components, their self-organization, emergent behavior, scale invariance, in addition to their adaptability, support the functional balance required for life (Rivera et al., 2020). In this context, proper functionality and adaptability are necessary to maintain health. Homeostasis is given by the balance between robustness, which define a system and its conformation, and adaptability (which is the ability of the system to respond to changes in the environment) while preserving its functionality (Fossion et al., 2018). Loss of this balance leads to disease. The systemic interactions within human physiology change as a function of many factors, age and sex being prominent examples. Here, by “sex” we refer to “the classification as male or female according to reproductive organs and functions assigned by the chromosomal complement” (Institute of Medicine (US) Committee on Understanding the Biology of Sex and Gender Differences, 2001). The expression of this sexual differentiation produces changes in many organ systems and across the lifespan of the individual, influencing how our bodies interact with the environment to determine health (Rich-Edwards et al., 2018). In this way, sex is recognized as a genetic modifier of disease pathophysiology, resulting in variations which should be considered in the biomedical enterprise (Mauvais-Jarvis et al., 2020).

There is ample evidence that the mechanisms underlying the regulation of various homeostatic processes are different in men and women. Physiologically, differences have been found in the function and morphology, for example, of the distribution and metabolism of adipose tissue (Palmer and Clegg, 2015), leading to the development of different types of obesity in women and men (Lumish et al., 2020). At the central nervous system level, morphological differences have been demonstrated in terms of structures and information processing: For instance, the thickness of the cortical mantle is greater in women than in men; also, memory consolidation is also different between sexes (Maren et al., 1994; Piefke et al., 2005). Other examples of physiological differences are specific pathologies such as neurodevelopmental disorders and neurodegenerative diseases, which are more frequent in men than in women (Hanamsagar and Bilbo, 2016), while on the other hand it is known that women have a greater predisposition to develop autoimmune diseases (Ngo et al., 2014), or gastrointestinal problems such as achalasia (Furuzawa-Carballeda et al., 2015; Rivera et al., 2021). The response to different pathogens also seems to depend on sex (McClelland and Smith, 2011). In the cardiovascular system, various studies provide evidence that men have a greater predisposition to develop cardiovascular diseases (Lloyd-Jones et al., 1999), greater cardiovascular mortality due to them (Regitz-Zagrosek, 2006), such that the risk factors between men and women are different. For example, while the average age at which women tend to experience myocardial infarction is higher than that of men, variables such as hypertension, diabetes and smoking result in significantly higher hazard ratios of myocardial infarction in women than in men (Mauvais-Jarvis et al., 2020). Metabolic syndrome may also develop differently due to physiological sexual dimorphism, as has been seen in other animals (Velasco et al., 2020).

**FIGURE 1:**
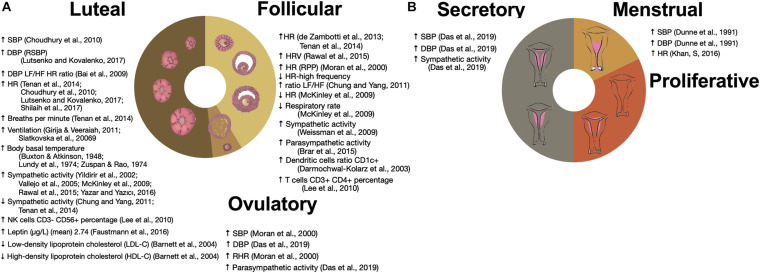
Influence of ovarian and menstrual cycles on physiological variables. **(A)** Changes in the time series variability and statistical distribution moments of physiological variables that have been associated with the ovarian cycle. **(B)** Changes in the time series variability and statistical distribution moments of physiological variables along the menstrual cycle. ↑ SBP (Choudhury et al., 2010), ↑ DBP (RSBP) (Lutsenko and Kovalenko, 2017), ↑ DBP LF/HF HR ratio (Bai et al., 2009), ↑ HR (Choudhury et al., 2010; Tenan et al., 2014; Lutsenko and Kovalenko, 2017; Shilaih et al., 2017), ↑ Breaths per minute (Tenan et al., 2014), ↑ Ventilation (Slatkovska et al., 2006; Girija and Veeraiah, 2011), ↑ Body basal temperature (Buxton and Atkinson, 1948; Lundy et al., 1974; Zuspan and Rao, 1974), ↑ Sympathetic activity (Yildirir et al., 2002; Vallejo et al., 2005; McKinley et al., 2009; Rawal et al., 2015; Yazar and Yazıcı, 2016), ↓ Sympathetic activity (Chung and Yang, 2011; Tenan et al., 2014), ↑ NK cells CD3– CD56+ percentage (Lee et al., 2010), ↑ Leptin (ug/L) (mean) 2.74 (Faustmann et al., 2016), ↓ Low-density lipoprotein cholesterol (LDL-C) (Barnett et al., 2004), ↑ High-density lipoprotein cholesterol (HDL-C) (Barnett et al., 2004), ↑ HR (de Zambotti et al., 2013; Tenan et al., 2014), ↑ HRV (Rawal et al., 2015), ↑ HR (RPP) (Moran et al., 2000), ↓ HR-high frequency ratio LF/HF (Chung and Yang, 2011), ↓ HR (McKinley et al., 2009), ↓ Respiratory rate (McKinley et al., 2009), ↑ Sympathetic activity (Weissman et al., 2009), ↑ Parasym pathetic activity (Brar et al., 2015), ↑ Dendritic cells ratio CD1c+ (Darmochwal-Kolarz et al., 2003), ↑ T cells CD3+ CD4+ percentage (Lee et al., 2010), ↑ SBP (Moran et al., 2000), ↑ DBP Das et al., 2019), ↑ RHR (Moran et al., 2000), ↑ Parasym pathetic activity (Das et al., 2019), ↑ SBP (Das et al., 2019), ↑ DBP (Das et al., 2019), ↑ Sympathetic activity (Das et al., 2019), ↑ SBP (Dunne et al., 1991), ↑ DBP (Dunne et al., 1991), and ↑ HR (Khan et al., 2016).

Sex-based differences in normal physiology, including those dependent on the endocrine system, are present and have been described in isolation for many physiological systems. However, to examine system-wide properties and interactions, new methodologies are necessary. Here we use time series analysis (Fossion et al., 2018) and network theory applied to data mining (Stephens et al., 2020) as two complementary approaches to study relations between physiological variables and their variability (Fossion et al., 2017). Cardiovascular coupling is a representative example of homeostatic regulation between different systems influenced by sex that is easily captured by several physiological time series. According to the control theory applied to homeostasis, physiological control systems are able to maintain the values of regulated variables that are vital for the organism within “normal” ranges because of the physiological responses of the corresponding effector variables which increase their variability (Fossion et al., 2018). In the cardiovascular system, heart rate serves as a regulatory variable and blood pressure as a regulated variable to sustain perfusion in the tissues. Different conditions and variations in metabolic activity induce changes in frequency and amplitude of the pulmonary and cardiac cycles in conjunction with vascular resistance and capacitance to provide an adequate constant oxygen supply. Modulation of these couplings is multidirectional in order to preserve homeostasis, and both acute and chronic diseases change this modulation through different pathophysiological mechanisms. This physiological modulation exhibits sex-based differences, and cyclic changes along the ovarian cycle phases (Figure 1). By examining differences in this modulation through time series analysis, different aspects of coupling are studied.

Sex differences are widespread across physiology, and may be classified as sex chromosome effects as well as hormonal effects that are either organizational or activational (Arnold et al., 2009). Therefore, physiological differences between men and women also occur in systems that are not easily recordable by continuous monitoring, and are instead widely approached through transversal studies of human populations. To take advantage of the wide selection of physiological variables available for transversal studies, correlation matrices in narrow-age cohorts can be used to construct complex inference networks (Hofer and Sliwinski, 2001; Batushansky et al., 2016; Barajas-Martínez et al., 2020, 2021; Cohen et al., 2021). Complex inference networks allow to find and explore statistical associations from the perspective of network theory, which provides a natural way to describe the relationships between a large set of entities (Stephens et al., 2018). Through networks, biological systems can be described by both graph-theoretical metrics as well as visual analysis (Aittokallio and Schwikowski, 2006; Merico et al., 2009; Pavlopoulos et al., 2011). This approach provides the possibility of observing the aggregate behavior of the system, offering insights on the function and structure of the system (Arnold et al., 2009; Jansson, 2020).

Historically, basic and clinical research has been carried out preferably in men or male animals. The bias to use male animals is based on reasons that have to do with the practicality of the study. For example, the preference for using male animals may be due to the fact that individuals are usually larger and therefore easier to manipulate when dissecting, or having better access in neurological studies for example. Another justification is that they do not have estrous cycles that strongly alter various physiological variables. There are also specific considerations such as the possibility of a differential response to treatment between men and women, and it is specifically for this reason that gender disparities should not be overlooked (Buoncervello et al., 2017). From preclinical studies (in animal models) to clinical studies, the study of the mechanisms involved in the appearance of many pathologies must consider the existence of different behavior and regulatory mechanisms between sexes, which is why there are explicit recommendations to include both sexes in preclinical studies (Sandberg et al., 2015) and to consider it in clinical treatment and evaluation (Mauvais-Jarvis et al., 2020). The aim of this study is to explore differences in the properties of the physiological systems of women and men using integrative approaches.

## Materials and Methods

The present contribution analyzes the differences that exist in physiological regulation mechanisms between women and men. To see these differences on one physiological variable (cardiac frequency) was studied by the time series obtained from electrocardiographic records of men and women from the Fantasia database of Physionet (Iyengar et al., 1996; Goldberger et al., 2000). To see systemic differences, a network analysis of biochemical and anthropometric biomarkers was carried out using a database of healthy men and women, using the physiological network model previously reported by our research group in Barajas-Martínez et al. (2021).

### Analysis of Cardiac Variability Between Women and Men Using the Fantasia Database

The Fantasia database (Iyengar et al., 1996) provided by Physionet (Goldberger et al., 2000) was used. This database was compiled by the Massachusetts Institute of Technology and the Beth Hospital in Israel (MIT/BIH DB), which is available at https://www.physionet.org/ (Iyengar et al., 1996; Goldberger, 1996). The database contains the 120 min electrocardiogram records of 10 men and 10 women between 21 and 34 years old, and 10 men and 10 women from 68 to 85 years old, in supine condition, while they were watching the Disney movie “Fantasia”.

To study heart rate variability, time series of intervals between successive QRS complexes (RR intervals) were used. The statistical moments of the resulting time series were calculated: arithmetic mean (μ), standard deviation (SD), skewness (sk), and kurtosis (k). The analysis was also carried out using non-linear measures, such as Shannon’s entropy, which measures the transfer of information (Pincus and Viscarello, 1992; Goldberger, 1996). Poincaré plots (obtained by plotting the time series against a version of it shifted by one time unit) were also studied, as they offer a measure of the first order correlations between the values in the series. The Poincaré plot is generally represented as an ellipse containing 95% of the points, the longitudinal diameter of this ellipse (SD1) describes the long-term deviation of the heart rate, and the cross-sectional diameter (SD2) represents short-term changes in heart rate. Student *t*-test was used to measure statistical differences between all the groups compared with the young women group.

### Sexual Differences in the Physiological Network

#### Ethics Statement

The study was developed according to Good Clinical Practice guidelines and the Declaration of Helsinki, and was carried out in accordance with current regulation contained in the Mexican Official Normativity, NOM-012-SSA3-2012. The Ethics Committee of the Facultad de Medicina of the Universidad Nacional Autónoma de México (UNAM) approved the procedures and protocols for this study as project FM/DI/023/2014. All the participants provided written informed consent.

#### Database

**FIGURE 2:**
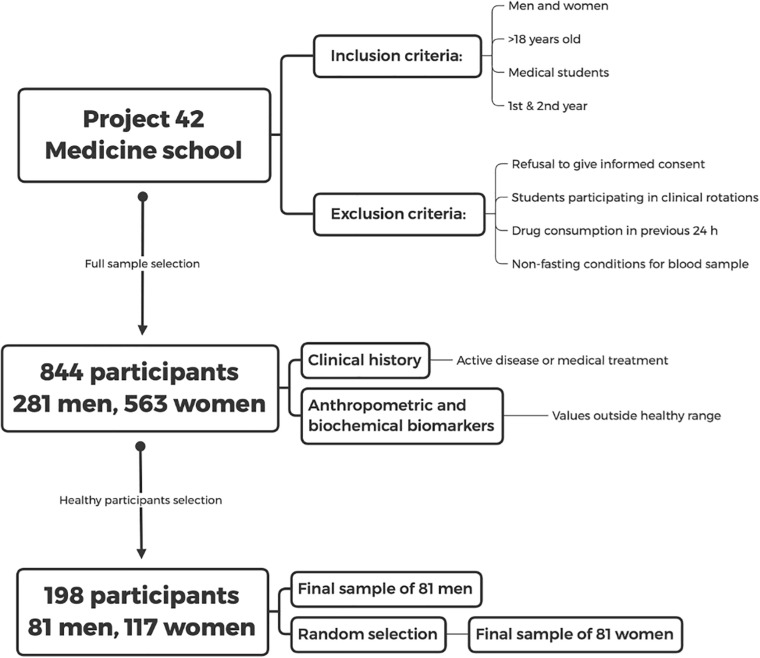
Study design. Inclusion and exclusion criteria for the database of the Medical School which was taken from the later database of “Project 42” are shown. The selection procedure of the healthy sample is described in the text.

The dataset employed for the study of physiological networks is available in Barajas-Martínez et al. (2021). We conducted a cross-sectional analysis of first- and second-year students living in Mexico City and its metropolitan surroundings. Inclusion criteria were men and women above 18 years old in the first and second years of the School of Medicine at UNAM. Exclusion criteria were refusal to give informed consent, students already participating in clinical rotations, drug consumption in the 24 h previous to the study and non-fasting at the time of blood sample collection (Figure 2).

#### Demographic Description of the Participants

After initially screening 844 participants, 281 men and 563 women, were included and underwent an extensive questionnaire, measurements of anthropometric variables, and evaluation of biochemical biomarkers. Sixty-nine percent of the participants were women, with an age ranging from 18 to 28 years old (mean age of 20 ± 2 years), reflecting the demographic composition of the School of Medicine. The detailed questionnaire included a brief medical history, evaluation of socioeconomic status, and health-related questions on sleep, exercise and dietary habits. Participants with active diseases or with any value outside the healthy ranges described in Tables 1, 2 and were classified as non-healthy. Specifically, after the normalization procedure described below in Eq. 2, rows were excluded if they contained any value above 1 or below 0. Only 198 participants, 81 men and 117 women, were selected as healthy. For the purpose of our analysis, health was defined as the absence of active disease, medication for chronic disease, and any physiological parameter outside the normal range. The aim of this operational definition is to select a very specific physiological state, where all possible indicators of health are within standard ranges. As such, this definition is not intended as a general-purpose definition of health.

**TABLE 1:** Criteria of health in men.

ID variable	Variable	Units	Low	High	Minimum	Maximum	*N*	Clinical criteria references
					
			Clinical criteria		Project 42 controls			
D0	Sex							
D1	Age	Years old			18	27	47	
P0	Systolic blood pressure (SBP)	mmHg	90	120	90	120	75	Whelton et al., 2018
P1	Diastolic blood pressure (DBP)	mmHg	60	80	60	80	76	Whelton et al., 2018
DP0	Pulse pressure (PP)	mmHg	20	60	30	60	75	Whelton et al., 2018
DP1	Mean arterial pressure (MAP)	mmHg	70	93	70	93	75	Whelton et al., 2018
T0	Axillary temperature	°C	35.5	37	35.2	37	26	Sund-Levander et al., 2002
T1	Tympanic temperature	°C	35.4	37.8	35.1	37.6	19	Sund-Levander et al., 2002
T2	Wrist temperature	°C		37.5	34.9	36.7	19	Holt et al., 2020
B0	Weight	kg			46.1	86.2	76	
B1	Height	m	1.6		1.53	1.85	76	Grimberg et al., 2016
DB1	Body mass index (BMI)	kg/m^2^	18	27	18	27.3	76	Wall-Medrano et al., 2016
B2	Waist	cm		90	63	93.5	75	Almeda-Valdes et al., 2016
B3	Hip	cm			77.5	111	75	Almeda-Valdes et al., 2016
B4	Arm circumference	cm	24		22	35	72	Tang et al., 2020
B6	Triceps	cm			2	30	76	
B7	Biceps	cm			0	40	76	
B8	Suprailiac	cm			0	36	75	
B9	Subscapular	cm			0	54	76	
DB2	Waist to hip ratio			0.95	0.72	0.92	75	Lear et al., 2010
DB3	Waist to height ratio			0.54	0.377	0.548	75	Almeda-Valdes et al., 2016
B10	Body fat	%			7.2	38	74	
B11	Body water	%			48.5	71	58	
DB4	Body fat	kg			3.5352	21.5352	74	Freedman et al., 2012
DB5	Total body water	kg			26.78	51.01	58	Chumlea et al., 2001
M1	Triglycerides	mg/dL	40	150	40	126	20	Mach et al., 2020
M2	Total cholesterol	mg/dL		200	109	200	20	Mach et al., 2020
M3	HDL cholesterol	mg/dL	40	90	41.6	67.3	8	Mach et al., 2020
M4	LDL cholesterol	mg/dL		116	58.2	109.7	8	Mach et al., 2020
M5	Glucose	mg/dL	70	100	70	94	20	Alberti et al., 2009
M6	Basal insulin	μUI/mL	2.6	24.9	2.5	9.04	20	
M7	Urea	mg/dL	10	50	19.26	48	20	Tyagi and Aeddula, 2020
M8	Blood urea nitrogen (BUN)	mg/dL	9	23	9	22.42	20	Tyagi and Aeddula, 2020
M9	Uric acid	mg/dL		6.8	3.27	6.81	20	Khanna et al., 2012
M10	Serum creatinine	mg/dL	0.8	1.5	0.82	1.2	20	Hosten, 1990
M12	Glycosylated hemoglobin (HbA1c)	%		5.7	4.7	5.8	20	American Diabetes Association, 2020
M13	C-reactive protein	mg/L	0	0.65	0.009	0.319	8	Oda et al., 2006
M17	Calcium	mmol/L	8.8	10.7	9.2	10.3	12	Shrimanker et al., 2019
M18	Phosphorus	mmol/L	2.3	6	2.6	4.4	12	
M19	Total bilirubin	mg/dL	0.2	1.3	0.51	0.95	12	Zhang et al., 2017
M20	Direct bilirubin	mg/dL	0	0.3	0.22	0.33	12	Zhang et al., 2017
M21	Indirect bilirubin	mg/dL	0.09	0.65	0.29	0.62	12	Zhang et al., 2017
M22	Aspartate aminotransferase	IU/L	5	35	18	28	12	Lala et al., 2020
DM0	Homeostasis model assessment of insulin resistance (HOMA IR)			1.7	0.488	1.708	20	Esteghamati et al., 2009
DM1	Estimated glomerular filtration rate (eGFR)	ml/min	90	120	85	126	16	Musso et al., 2016
DM2	Estimated average glucose (eAG)	mg/dL			88.19	119.76	20	Guo et al., 2020
DM3	eAG– fasting glucose	mg/dL			5.06	31.76	20	Guo et al., 2020
DM4	BUN to creatinine ratio				8.17	21.69	20	Haines et al., 2019
H0	Leukocytes	10^9^/L	3.5	12	4.4	8.5	13	Leung et al., 2020
H1	Neutrophils	10^9^/L	1.9	8	1.99	5.92	13	Coates et al., 2020
DH1	Total neutrophils percentage	%	40	70	40	70	13	Coates et al., 2020
H2	Segmented neutrophils percentage	%			39.7	69.6	11	
H3	Lymphocytes	10^9^/L	1.5	3	1.44	3.25	12	Rosenthal, 2020
DH2	Lymphocytes percentage	%	20	40	23.1	47.34	12	Rosenthal, 2020
H4	Monocytes	10^3^/L	0.16	1	0.24	0.68	13	Steensma and Bolton, 2020
DH3	Monocytes percentage	%	0	10	4	10.01	13	Steensma and Bolton, 2020
H5	Eosinophils	10^3^/L	0	0.8	0.01	0.33	13	Rosenthal, 2020
DH4	Eosinophils percentage	%	0	5	0.1	5.1	13	Rosenthal, 2020
H6	Basophils	10^3^/L	0	0.2	0.01	0.199	13	Rosenthal, 2020
DH5	Basophils percentage	%	0	1	0.1	1.2	12	Rosenthal, 2020
H7	Erythrocytes	10^12^/L	4.6	6.2	4.8	6.1	13	Adeli et al., 2015
H8	Hemoglobin	g/dL	14.9	18.7	14.8	17.9	13	Adeli et al., 2015
H9	Hematocrit	%	40	54	43.2	54.1	13	Adeli et al., 2015
H10	Mean corpuscular volume	fL	76	100	79.1	96	13	Leung et al., 2020
H11	Mean concentration of hemoglobin	pg/RBC	27.5	33.2	25.5	31.8	13	Leung et al., 2020
H12	Mean corpuscular hemoglobin concentration	g/dL	32.5	35.2	31.5	33.9	11	Leung et al., 2020
H13	Red cell width distribution	%	11.4	13.5	12.3	14.6	11	Leung et al., 2020
H14	Platelets	10^3^/μL	147	384	159	340	13	Leung et al., 2020
H15	Mean platelet volume	fL	6	13.2	6.1	10.3	11	Leung et al., 2020

**TABLE 2:** Criteria of health in women.

ID variable	Variable	Units	Low	High	Minimum	Maximum	*N*	Clinical criteria references
					
			Clinical criteria		Project 42 controls			
D0	Sex							
D1	Age	Years old			17	28	81	
P0	Systolic blood pressure (SBP)	mmHg	90	120	90	120	101	Whelton et al., 2018
P1	Diastolic blood pressure (DBP)	mmHg	60	80	58	80	101	Whelton et al., 2018
DP0	pulse pressure (PP)	mmHg	20	60	12	40	101	Whelton et al., 2018
DP1	Mean arterial pressure (MAP)	mmHg	70	93	70	93	101	Whelton et al., 2018
T0	Axillary temperature	°C	35.5	37	35.4	37.3	55	Sund-Levander et al., 2002
T1	Tympanic temperature	°C	35.4	37.8	35.5	37.1	46	Sund-Levander et al., 2002
T2	Wrist temperature	°C		37.5	34.4	36.8	45	Holt et al., 2020
B0	Weight	kg			43.7	73.2	102	
B1	Height	m			1.45	1.73	100	Grimberg et al., 2016
DB1	Body mass index (BMI)	kg/m^2^	18	27	17.5	27.2	100	Wall-Medrano et al., 2016
B2	Waist	cm		80	60	82.5	100	Almeda-Valdes et al., 2016
B3	Hip	cm			76	111	102	Almeda-Valdes et al., 2016
B4	Arm circumference	cm	24		20	32	100	Tang et al., 2020
B6	Triceps	cm			5	31	102	
B7	Biceps	cm			0	28	102	
B8	Suprailiac	cm			1	34	102	
B9	Subscapular	cm			2	37	102	
DB2	Waist to hip ratio			0.8	0.67	0.85	100	Lear et al., 2010
DB3	Waist to height ratio			0.54	0.38	0.53	98	Almeda-Valdes et al., 2016
B10	Body fat	%			11.3	47.9	102	
B11	Body water	%			30.5	64.7	64	
DB4	Body fat	kg			5.7	29.87	102	Freedman et al., 2012
DB5	Total body water	kg			15	36.1	64	Chumlea et al., 2001
M1	Triglycerides	mg/dL	40	150	44	150	46	Mach et al., 2020
M2	Total cholesterol	mg/dL		200	106	202	46	Mach et al., 2020
M3	HDL cholesterol	mg/dL	50	90	40.9	68.8	25	Mach et al., 2020
M4	LDL cholesterol	mg/dL		116	50.2	112	25	Mach et al., 2020
M5	Glucose	mg/dL	70	100	66	90	46	Alberti et al., 2009
M6	Basal insulin	μUI/mL	2.6	24.9	2.73	9.39	47	
M7	Urea	mg/dL	10	50	19	40.66	46	Tyagi and Aeddula, 2020
M8	Blood urea nitrogen (BUN)	mg/dL	9	23	9	19	46	Tyagi and Aeddula, 2020
M9	Uric acid	mg/dL		6.8	1.9	6.9	45	Khanna et al., 2012
M10	Serum creatinine	mg/dL	0.5	1.1	0.55	0.94	46	Hosten, 1990
M12	Glycosylated hemoglobin (HbA1c)	%		5.7	4.6	5.6	46	American Diabetes Association, 2020
M13	C-reactive protein	mg/L	0	0.65	0.009	0.544	24	Oda et al., 2006
M17	Calcium	mmol/L	8.8	10.7	8.6	10.1	20	Shrimanker et al., 2019
M18	Phosphorus	mmol/L	2.3	6	3.7	4.8	21	
M19	Total bilirubin	mg/dL	0.2	1.3	0.2	0.97	21	Zhang et al., 2017
M20	Direct bilirubin	mg/dL	0	0.3	0.1	0.31	21	Zhang et al., 2017
M21	Indirect bilirubin	mg/dL	0.09	0.65	0.09	0.66	21	Zhang et al., 2017
M22	Aspartate aminotransferase	IU/L	5	35	12	25	21	Lala et al., 2020
DM0	Homeostasis model assessment of insulin resistance (HOMA IR)			1.8	0.5	1.83	21	Esteghamati et al., 2009
DM1	Estimated glomerular filtration rate (eGFR)	ml/min	90	120	89	124.3	30	Musso et al., 2016
DM2	Estimated average glucose (eAG)	mg/dL			85.32	114.02	46	Guo et al., 2020
DM3	eAG– fasting glucose	mg/dL			5.93	114.02	46	Guo et al., 2020
DM4	BUN to creatinine ratio				10.34	32	46	Haines et al., 2019
H0	Leukocytes	10^9^/L	3.5	12	4.6	10.5	23	Leung et al., 2020
H1	Neutrophils	10^9^/L	1.9	8	1.9	7.03	23	Coates et al., 2020
DH1	Total neutrophils percentage	%	40	70	41.25	70.21	23	Coates et al., 2020
H2	Segmented neutrophils percentage	%			41.25	72.1	20	
H3	Lymphocytes	10^9^/L	1.5	3	1.48	3.42	23	Rosenthal, 2020
DH2	Lymphocytes percentage	%	20	40	19.9	49.1	23	Rosenthal, 2020
H4	Monocytes	10^3^/L	0.16	1	0.16	0.74	23	Steensma and Bolton, 2020
DH3	Monocytes percentage	%	0	10	1.9	9.6	23	Steensma and Bolton, 2020
H5	Eosinophils	10^3^/L	0	0.8	0.03	0.31	21	Rosenthal, 2020
DH4	Eosinophils percentage	%	0	5	0.02	4.5	23	Rosenthal, 2020
H6	Basophils	10^3^/L	0	0.2	0.01	1.03	23	Rosenthal, 2020
DH5	Basophils percentage	%	0	1	0.16	1.03	23	Rosenthal, 2020
H7	Erythrocytes	10^12^/L	4.2	5.4	4.2	5.42	23	Adeli et al., 2015
H8	Hemoglobin	g/dL	12	16	13.2	16.3	23	Adeli et al., 2015
H9	Hematocrit	%	36	48	40.1	48.9	23	Adeli et al., 2015
H10	Mean corpuscular volume	fL	76	100	82.4	100.7	23	Leung et al., 2020
H11	Mean concentration of hemoglobin	pg/RBC	27.5	33.2	27.5	33.3	23	Leung et al., 2020
H12	Mean corpuscular hemoglobin concentration	g/dL	32.5	35.2	29.8	34	20	Leung et al., 2020
H13	Red cell width distribution	%	11.4	13.5	12.1	14.5	20	Leung et al., 2020
H14	Platelets	10^3^/μL	147	384	148	377	23	Leung et al., 2020
H15	Mean platelet volume	fL	6	13.2	5.9	12	20	Leung et al., 2020

We found no differences between men and women in current consumption of soft drinks and snacks. Self-reported current, previous year and 5 years ago sleep and exercise time were compared by mixed-effects analysis with Tukey’s *post hoc* test for effects within rows and columns. Both men and women had significant reductions in the time of weekly exercise, *F*(2,2246) = 80.4, *p* < 0.001, and daily sleep, *F*(1.97,1474) = 480, *p* < 0.001, compared to the time invested in these activities previously. Men reported more current weekly exercise (Median = 3 h) than women (Median = 1 h). This difference was statistically significant, U(59, 107) = 2075, *p* < 0.0001. However, these values were not statistically different from the values of their sex-matched unhealthy counterparts (Median = 2 h, *p* = 0.55 for unhealthy men and median = 1 h, *p* = 0.97 for unhealthy women). Thus, differences between men and women would be the result of physiological differences between sexes and not due to dietary habits or exercise. Even within healthy ranges, body weight, height, waist circumference, body fat and body water, arm circumference, triceps skinfold width, systolic and mean blood pressure, creatinine, uric acid, erythrocytes, hemoglobin, and hematocrit were significantly different between men and women in multiple Mann–Whitney tests with Bonferroni-Dunn correction for multiple comparisons (see Supplementary Table 2). The full database including health questionnaires is available at https://www.c3.unam.mx/health/.

#### Measurement of Physiological Variables

All samples and anthropometric measurements were obtained in fasting conditions from 7:00 to 9:00 a.m. after a medical check-up. Anthropometric measurements and vital signs were taken using following established protocols by trained medical personnel. A total of 50 variables related to anthropometry, bioimpedance, hematic biometry, and blood chemistry were assessed (see list of variables in Tables 1, 2). The database also comprises 12 derived variables widely used to define meaningful relationships between variables (formula to evaluate derived variables are given on Supplementary Table 1). The dataset used for the methodology and data analysis is available in Barajas-Martínez et al. (2021). Healthy men and women individuals were selected from the original sample by clinical diagnosis by medical doctors, clinical history, and using thresholds established in the literature to differentiate between normal values and abnormal values (Tables 1, 2). Based on current medical understanding, these thresholds are not meant as a diagnostic of illness, but rather are sufficiently stringent to suggest the increased risk of an individual to contract a disease. After screening using these healthy control, two groups were selected: one of 81 men, and another of 117 women (databases available on Supplementary Material). To ensure parity on size between groups 81 women were chosen at random from the 117 original samples. Characteristics of all anthropometric and biochemical measurements are displayed for men (see Table 1) and women (see Table 2). There is a comprehensive summary of the biochemical procedures available (Barajas-Martínez et al., 2020). The formulas employed for the calculation of derived variables are accessible in the Supplementary Table 1.

### Data Processing

Statistical moments were calculated for all variables in order to describe fine details of their variability within the included populations. Calculations were carried out using Excel^®^ and Origin^®^ Pro 2020 and included the arithmetic mean (μ), standard deviation (SD), skewness (sk), and kurtosis (k). Using the statistical moments, the momentum space metric in SD-sk-κ space that indicates deviation from a Gaussian distribution was evaluated through (Rivera et al., 2016):

$$\alpha =\sqrt{{\left(\frac{S\,D}{\mu }\right)}^{2}+s\,k^{2}+k^{2}}$$

For a Gaussian distribution, $\alpha =\frac{S\,D}{\mu }$. The reduction in standard deviation (increase in rigidity), skewness with respect to the median (symmetry measure), and kurtosis (change to more leptokurtic distributions) indicates increased rigidity of the physiological variable (Rivera et al., 2016).

For the generation of the correlation matrix of physiological variables we use a novel approach. Instead of relying on the data distribution of the limited observations of a study, whenever possible, we use the normal ranges and criteria reported in clinical guidelines and medical references (Tables 1, 2). This allows for immediate identification of values above (>1) or below (<0) the thresholds set on the best evidence present in the literature. Incidentally, some physiological variables are expected to be displaced from these international normal ranges, for example, blood hemoglobin levels are increased in individuals living in cities at high altitude. In these cases, normal ranges previously reported for these special circumstances were employed.

For each variable from the data, we obtained the normalized value *X*_*i*_ applying the following normalization to the original data:

$$X_{i}=\left(\frac{V_{i}-M\,i\,n}{M\,a\,x-M\,i\,n}\right)$$

where:

$$V_{i}=d\,a\,t\,a\,v\,a\,l\,u\,e,$$

M⁢i⁢n=M⁢i⁢n⁢i⁢m⁢u⁢m⁢v⁢a⁢l⁢u⁢e⁢o⁢f⁢t⁢h⁢e⁢n⁢o⁢r⁢m⁢a⁢l⁢r⁢a⁢n⁢g⁢e,

M⁢a⁢x=M⁢a⁢x⁢i⁢m⁢u⁢m⁢v⁢a⁢l⁢u⁢e⁢o⁢f⁢t⁢h⁢e⁢n⁢o⁢r⁢m⁢a⁢l⁢r⁢a⁢n⁢g⁢e.

There are no applicable ranges or health guidelines in the literature for certain physiological variables. If this is the case, after exclusion of all unhealthy individuals, the range of data values was used for normalization.

### Network Construction

The construction of complex inferential networks of this contribution employed and replicated the same methods as previously presented in Barajas-Martínez et al. (2020, 2021) unless otherwise specified. After the normalization procedure, each variable was identified with a node number as indicated in Tables 1, 2. The Spearman rank correlation ρ was selected as a suitable measure of correlation for the generation of the correlation matrix between physiological variables (Batushansky et al., 2016). The Spearman rank correlation is a non-parametric measure of the statistical dependency between the rank values of the variables considering a monotonic relationship (not necessarily linear) and is not influenced by normalization procedures. When missing values were present, we performed pairwise deletion without data imputation in order to use all available participants who provide data relevant for each correlation. For each pair of physiological variables, X, Y, rank (*rk*_*X*_,*rk*_*Y*_, respectively), standard deviation (*SD*_*rkX*_*SD*_*rkY*_) were evaluated, and the Spearman rank correlation was calculated as the ratio between covariance (cov) and deviations:

$$\rho =\frac{c\,o\,v\,\left(r\,k_{X},r\,k_{Y}\right)}{S\,D_{r\,k\,X}\,S\,D_{r\,k\,Y}}$$

A Student’s *t* distribution with (n-2) degrees of freedom was employed to test the Spearman rank correlation and the significance of its departure from zero. A *p*-value threshold was selected for best connectedness and modularity (Barajas-Martínez et al., 2021). In this case, significant correlations were defined using a threshold value of *p* < 0.05, indicating that the relation does not support the null hypothesis that the independent and dependent variables are unrelated. For the construction of this Spearman correlation matrix, Prism 8.1.2 (277), GraphPad^®^ Software, La Jolla, CA, United States, www.graphpad.com was employed. After filtering the correlation matrix by the *p*-value threshold (*p* < 0.05), the ρ coefficients were squared in order to obtain only positive values between pairs of physiological variables, generating the adjacency matrix of the network. The resulting network is weighted and undirected. For the network construction RStudio^®^, an R^®^ language programming suite and igraph package were employed (Csárdi et al., 2016; R Core Team, 2020; RStudio Team, 2020).

In short, nodes within a network can be ranked according to several interpretations of importance that fall into two different categories, radial and medial measurements (Borgatti and Everett, 2006). In this work, we used eigencentrality as a measure of influence within the network and betweenness as the measure of intermediation. This centrality indicates either the influence of nodes (radial measure) or gatekeeping (medial measure) within the network. Centrality values were obtained using the evcent and betweenness functions from the SNA package. Assortativity of these centralities, i.e., the propensity of nodes with similar centrality to link together, was calculated. To assess whether networks had overall different centrality values, Wilcoxon’s matched-pairs signed test were done. Spearman rank correlation was done to determine whether centrality values of the nodes were correlated in both networks. To showcase hierarchical arrangement from the node with highest eigencentrality, Sugiyama layout was implemented (Sugiyama et al., 1981). Nodes were placed into layers to minimize crossings. Since our networks contain cycles, the weakest links in the cycle was broken first.

### Cluster Detection

Three different approaches were used to ascertain which physiological variables are more closely related within the system. First, we employed a robust approach to Principal Component Analysis using our correlation matrix, then, for the networks we used clustering algorithms and topological clusters. Principal Component Analysis was applied to the Spearman correlation matrix, then physiological variables were ordered according to the angular positions of their eigenvectors using the first and second principal components, placing the most similar variables contiguously. The order of the correlation matrix of women was selected to allow side by side comparison between matrices, as this system was more resilient.

To ascertain whether a natural aggregation of physiological variables is present in the networks, clustering algorithms were used, which is an advantage of network approaches that are not available in reductionist methods. Modularity was used to test whether these groupings fulfilled the requirement to exceed the internal edge density that would be expected in an analogous network where connections are arranged at random (Newman, 2006). The cluster detection maintained and replicated the same methodology described in Barajas-Martínez et al., 2021. In the literature, two types of clustering are recognized, topological clusters according to the placement of nodes within an energy model layout, and communities defined by appropriate algorithms grouping the vertices within a graph such that they are more densely linked to each other than to other vertices (Csárdi et al., 2016). As in the cited article, energy-model layouts such as a Linear logarithmic layout (Linlog) identify topological clusters that complement the representation of the community structure of a network (Noack, 2009; Mihaicuta et al., 2017). In this layout, distance is independent of path length, thus nodes with high collinearity are simply overlapped. For this contribution two different clustering algorithms included in the igraph package were tested, Louvain (Blondel et al., 2008) and Spinglass (Reichardt and Bornholdt, 2006). The Louvain algorithm is a heuristic method based on modularity optimization, implemented in the cluster_louvain function from the igraph package (Blondel et al., 2008). The spinglass community algorithm selects those nodes with the greatest probability to be found in the same state concurrently, with the cluster_spinglass function from the igraph package (Reichardt and Bornholdt, 2006). The results of this automated clustering were then examined against previous research with the aim of finding functional systems that best described the nodes. Various measures of clustering comparison were employed to evaluate the degree of correspondence between the two networks, namely, variation of information, normalized mutual information, and Rank Index (Rand, 1971; Meilǎ, 2003; Danon et al., 2005). In order to produce a cluster network showing the interactions between various functional clusters, as previously stated, all nodes within the same cluster were contracted into the node of greatest eigencentrality (Barajas-Martínez et al., 2021).

### Topological Properties

In order to test whether overall differences in topology were indeed present, 30 networks for men and 30 networks for women were assembled from correlation matrices that comprehended only 60 individuals selected at random each time from the original 81 healthy men and 117 healthy women. To study network resilience, and vulnerability, the NetSwan package was used. Differences between networks were tested with a paired Friedman’s test and Dunn’s *post hoc* test. The topological properties were evaluated as follows: the density of the network, reciprocity and characteristic path length were calculated using the igraph package. The DirectedClustering package was used for the calculation of the weighted transitivity and the clustering coefficient of these undirected weighted networks (Clemente and Grassi, 2018). As calculated by qgraph the small-world index, a measure that describes the relation between the clustering coefficient and the mean pathlength in a network against what would be expected in a random network, was used as a summary metric of the network topology (Watts and Strogatz, 1998).

## Results

Significant differences on the physiology of men and women were found using two complementary analysis: a univariate physiological time series study carry-on in heart rate variability (section “Analysis of Heart Rate Variability Using the Physionet Fantasia Database”) and a systemic approach by a physiological network construction (section “Physiological Network”).

### Analysis of Heart Rate Variability Using the Physionet Fantasia Database

**FIGURE 3:**
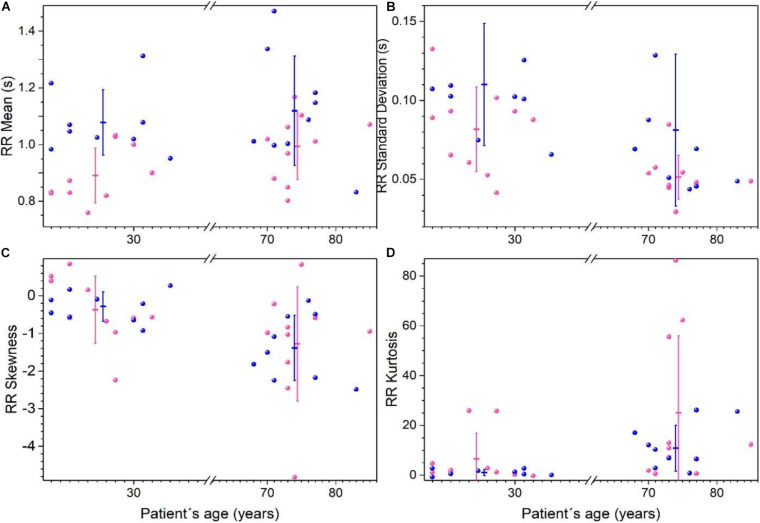
Statistical moments of time series of the RR heartbeat intervals from Physionet’s Fantasia database. Each point corresponds to data from a women (pink) or men (blue) subject. For each group (women, men, young, and old) the mean of each parameter ± the standard deviation is plotted for RR average **(A)**, standard deviation **(B)**, skewness **(C)**, and kurtosis **(D)**. (Adapted from Lavin-Perez and Rivera, 2018).

Heart rate records from Fantasia database were analyzed using the time intervals between R peaks on the electrocardiogram (RR) considering four groups: young women, young men, old women, and old men. Statistical moments are shown in Figure 3, and reported in Supplementary Table 3. RR mean values are higher for young men than for young women (*p* < 0.05), while for older men and women no differences are found, both groups have tachycardic records (see Figure 3A). Variability of RR (measure by the standard deviation) is higher on young men compare young women, and older women have more robust heart rate because they show the lowest variability of all the groups (see Figure 3B). RR distributions are not symmetric as shown in skewness plot (Figure 3C). With respect to the skewness, there are no differences between men and women, however, RR is more asymmetric for older subjects. Additionally, there are significant changes observed between sexes in kurtosis, but for older subjects the RR distributions are leptokurtic (see Figure 3D).

**FIGURE 4:**
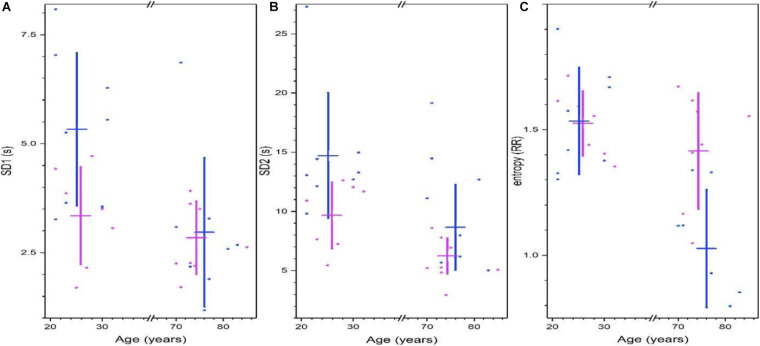
Non-linear analysis of heart rate variability from Physionet’s Fantasia database. Parameters of the 95% Poincare’s ellipse of the data SD1 **(A)** and SD2 **(B)**. Shannon’s entropy is shown in **(C)**. For all graphics. Each point corresponds to data from a women (pink) or men (blue) subject, while vertical lines correspond to the mean ± the standard deviation for each group (women, men, young, and old). (Adapted from Lavin-Perez and Rivera, 2018).

Poincare’s plots are shown in Figure 4, Shannon’s entropy and measures of the 95% ellipse (SD1, SD2, and eccentricity) are given in Supplementary Table 4. For young subjects, RR short-range correlations measure by Poincare’s ellipse SD1 is greater in men, and this variability is reduced with age, for old subjects, there is no sex difference (Figure 4A). The long-term deviation of the RR time series measure by Poincare’s ellipse SD2 is greater in young men, and is reduced in older men (Figure 4B). Shannon’s entropy is shown as a complementary approach to characterize the variability of heart rate. Interestingly, this measure is similar in young men and women, and remains high for older women, but is decreased for older men (Figure 4C).

### Physiological Network

**FIGURE 5:**
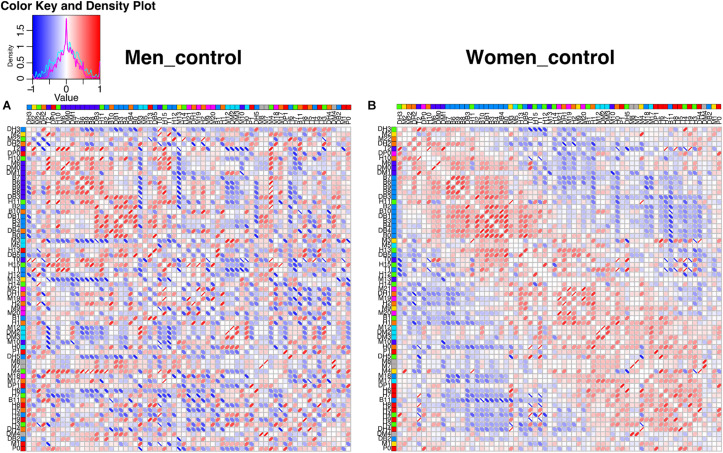
Unfiltered correlation matrix of men and women. Spearman correlation matrices are shown as heatmaps for men **(A)** and women **(B)**. The density plot for the correlations is presented in the upper left side, for men (cyan) and for women (magenta). Columns and rows are ordered according to the angular order of the eigenvectors of women matrix. The network clustering is shown in colors to the right of the labels of the physiological variables. Spearman correlations are presented as ellipses. Positive correlations are shown in shades of red while negative correlations are shown in shades of blue.

**FIGURE 6:**
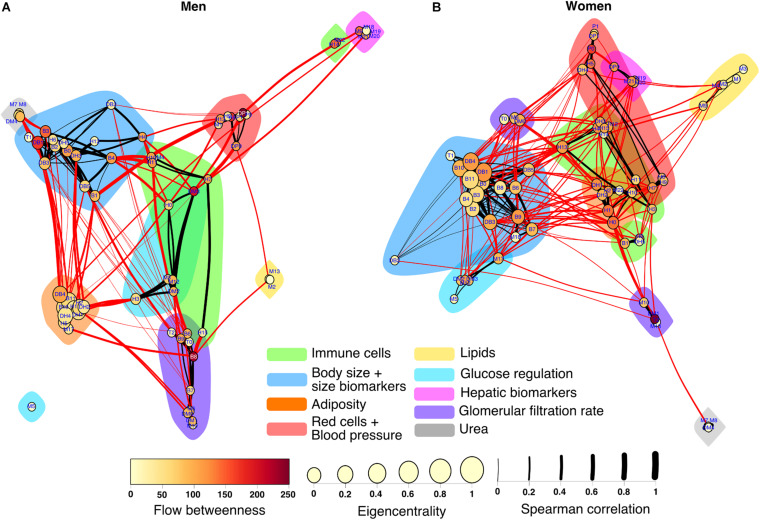
Physiological networks of men and women. The physiological network of men **(A)** and women **(B)** are represented using a Linlog force-directed model. Node size indicates the eigencentrality and node color the flow betweenness. Color clouds show nodes clustered together using the Louvain algorithm. Link width represents the strength of the Spearman correlation between physiological variables. Intercluster links are highlighted in red, whereas intracluster links are black.

**FIGURE 7:**
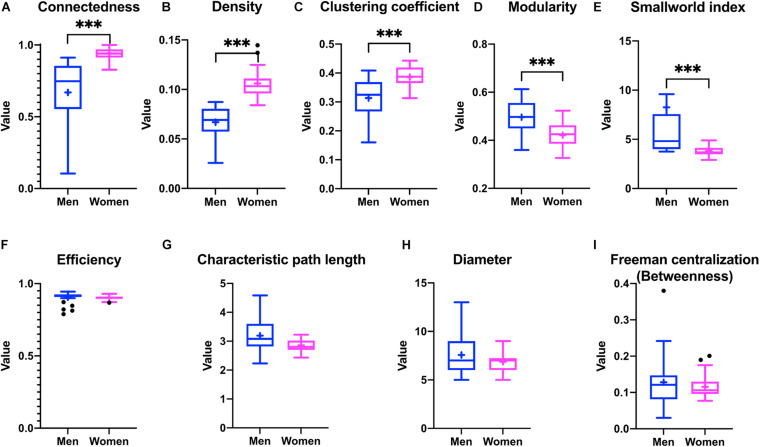
Differences in the topological characteristics of the networks for men and women. Differences in connectedness **(A)**, density **(B)**, clustering coefficient **(C)**, modularity **(D)**, smallworld index **(E)**, efficiency **(F)**, characteristic path length **(G)**, diameter **(H)**, and betweenness Freeman centralization **(I)** are shown between the physiological networks of men and women. Statistical significant difference is indicated by ^∗∗∗^ if *p* < 0.001.

The construction of physiological networks based on the Spearman correlation analysis provides characteristics that describe the regulation of physiological systems. They indicate the degree of robustness that is established in the relationship between different variables, and their grouping. As seen in the unfiltered correlation matrix, networks of men had a tendency to display stronger positive and negative correlations, and higher modularity, while women had less modularity (Figure 5). After applying the threshold, the topology shows the type of connections, their density and whether they are within-cluster (intra) or between-clusters (inter), which is of particular interest. Different physiological networks result for men and women (Figure 6). The resulting topological characteristics for these networks are summarized in Table 3. The size of these networks is fixed to the number of physiological variables surveyed. The degree distributions of the network of men and women were significantly different by Kolmogorov-Smirnov test (*D* = 0.4478, *p* < 0.0001). Connectedness was higher in the network of women, while efficiency was similar for both networks (Figures 7A,F). The physiological network of women had more edges and consequently greater density (Figure 7B). On the other hand, the characteristic path length, diameter and Freeman centralization for betweenness, all measures that depend on the path structure of the network, were similar. The average clustering coefficient is greater in the network of women and the differences add up to result in a greater small-world index in men (Figure 7). Consequently, modularity was higher in men than in women (Figure 7D). These characteristics provide information on the mechanism and regulation of a physiological system, suggesting that the physiological networks of men, being more small-world, are consequently more adaptable. These topological differences are reflected in the centrality measures of the nodes. Particularly, the nodes in the network of men had greater eigencentrality values than in the network of women (*W* = −690, *p* < 0.05), but betweenness values remained comparable in both networks (*W* = 92, *p* = 0.7). Regardless of these differences between networks, there was a significant correlation of eigencentrality (ρ = 0.61, *p* < 0.0001), and betweenness (ρ = 0.42, *p* < 0.001). This indicates that the role of each physiological variable either as an influence or as an intermediary remained similar both in men and women. When comparing clusters between the networks of men and women there was similarity (Table 4). All these elements suggest that while topological structure is quite different, the role of physiological variables and their association within the network remains similar.

**TABLE 3:** Topological characteristics of the physiological network for men and women.

	Men		Women		Mann-Whitney *U*	*P-*value
	Mean	SD	Mean	SD		
Connectedness	0.60	0.26	0.94	0.05	0	<0.000001
Density	0.06	0.02	0.10	0.01	1	<0.000001
Clustering coefficient	0.31	0.07	0.39	0.04	30	0.000323
Modularity	0.48	0.08	0.42	0.04	60	0.029496
Small world index	10.72	14.33	3.80	0.39	25	0.000113
Efficiency	0.89	0.05	0.90	0.01	83	0.232812
Characteristic path length	3	0.62	3	0.22	97	0.539299
Diameter	7	2.03	7	1.16	104	0.740211
Freeman centralization (Betweenness)	0.11	0.08	0.12	0.04	94	0.460959

**TABLE 4:** Comparison of clusters in the physiological networks for men and women.

	Spinglass			Louvain		
	Median	Maximum	Minimum	Median	Maximum	Minimum
Variation of information	2.18	2.42	1.98	2.59	2.59	2.59
Rand Index	0.82	0.84	0.79	0.76	0.76	0.76
Normalized mutual information	0.48	0.55	0.40	0.30	0.30	0.30

**FIGURE 8:**
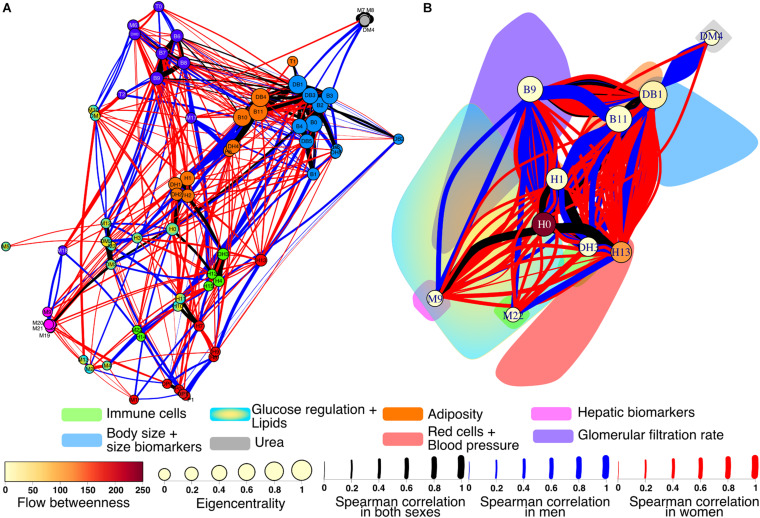
Differential physiological network. To showcase the differences between men and women, the physiological networks were superimposed onto an undirected network **(A)**. Links found in both networks are black, while blue links are present only in men and red only in women. The width of the links represents the strength of the correlation between physiological variables. Nodes are colored according to the clusters. For the cluster network **(B)** all nodes within the same cluster were contracted into the node of greatest eigencentrality. The color cloud represents the original area of the cluster. The eigencentrality and flow betweenness of each cluster are also shown.

Of particular interest for this work is to observe the differences between a typical network of healthy women versus healthy men. A differential network highlighting the presence or absence of each edge in the networks shows how interactions between physiological variables and clusters contrast (Figure 8). There 90 robust links that are present in both men and women from a total of 426. These correlations between physiological variables were strong and independent of sex. The physiological network for women has greater density, which appears readily in the larger number of exclusive links (221) than for men (115). However, there was no statistical difference between the strength of the nodes in the physiological network of men and women (*W* = −240, *p* = 0.5).

Several differences were apparent in the network and the cluster network (Figure 8). For instance, the urea/creatinine ratio (DM4) cluster is closely related to the body mass index, BMI (DB1) cluster only in men, while in women it is related to estimated glomerular filtration rate (DM1) node. Insulin (M6) and HOMA index (DM0) were correlated with serum creatinine (M10) and estimated glomerular filtration rate (DM1) in men, but not in women. In men, blood pressure related variables (P0, P1, DP0, and DP1) are strongly related to erythrocytes (H7), hemoglobin (H8), and hematocrit (H9). In men, plycometric measurements of tricipital (B5), bicipital (B6), suprailiac (B7), subscapular (B8) skinfolds are correlated with metabolic variables HDL cholesterol (M3), glycosylated hemoglobin HbA1c % (M12), and estimated average glucose (DM2), but not in women. Conversely, variables within the body fat (B9) cluster are related to neutrophil variables, total neutrophils (H1), segmented neutrophils (H2), neutrophils percentage (DH1), and segmented neutrophils percentage (DH2) only in women. As shown in the cluster network, the red cell distribution width (H13) cluster has plenty of connections with the BMI (DB1) cluster only in women. For men, several connections are present between the body water (B10) and the total neutrophils (H1) clusters, relating bioimpedance measurements body fat % (B9), body water % (B10), and body fat in kg (DB4) with the neutrophil variables H1, H2, DH1, and DH2. Finally, the neutrophils H1 cluster is connected in women with the uric acid (M9) cluster that also comprises bilirubin variables, total (M19), direct (M20), and indirect (M22).

**FIGURE 9:**
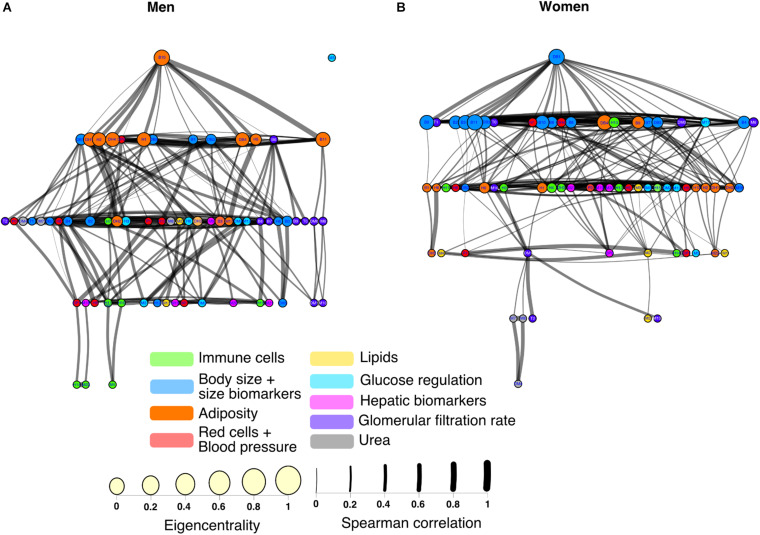
Hierarchy of physiological networks of men and women. The corresponding physiological networks are laid out hierarchically with the Sugiyama algorithm for men **(A)** and women **(B)**. Nodes are ordered into layers according to eigencentrality and placed to minimize crossings. The color of the node indicates the cluster to which it belongs in the network.

When arranged hierarchically with the Sugiyama layout, nodes with the greatest eigencentrality were body water % (B10) for men and BMI (DB1) for women (Figure 9). From these central nodes, all others can be reached in 4 steps for men and 5 steps for women, as shown by the number of layers, with the exception of glucose (M5) which is an isolated node for the network of men. There was a significant correlation between the hierarchical arrangement of both networks (ρ = 0.43, *p* < 0.001), indicating that the physiological variables were organized similarly in men and women. For the network of men, the nodes with greatest betweenness were subscapular (B8) and mean arterial blood pressure (DP1), while for women it was BMI (DM1). Assortativity is also apparent in this layout where nodes with the greatest eigencentrality are closer both in men (0.62) and women (0.47). Overall anthropometric physiological variables had both greater eigencentrality and are higher in the hierarchy than all other variables in both networks. Interestingly, some variables related to immune cells measured in the hematic biometry are peripheric in men, but more deeply located in the network of women.

**FIGURE 10:**
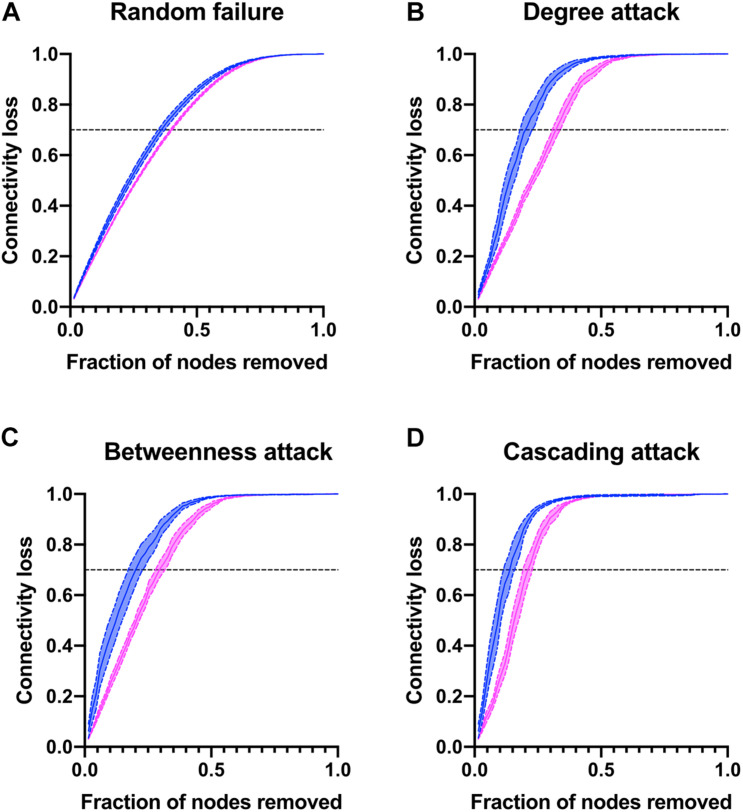
Network vulnerability to directed attacks. The connectivity loss is plotted against the number nodes removed either at random **(A)** or as a directed attack guided by degree **(B)**, betweenness **(C)** or cascading **(D)** attacks for men and women.

Finally, to better describe the network strengths and weaknesses, nodes were removed either at random or as a directed attack guided by degree, betweenness, or cascading (Figure 10). Characteristic of complex networks, directed attacks resulted in greater loss of connectivity than random removal of nodes. There were no differences in susceptibility to random failure between men and women, as expected. However, the physiological network of men was more vulnerable to directed attacks than the network of women. For instance, in order to obtain a loss of 70% of the network connectivity in a cascading attack, it is only required to remove 13% of the nodes for men while 21% are required for network of women. Likewise, in degree and betweenness attacks the network of men loses 70% of its connectivity with the removal of 19% of nodes, while network of women requires 30% removal. This is a result of the reduced density of the physiological network of men and how nodes with greater degree are placed within the network. Taken together, all these measures suggest that the physiological system is more resilient in women than in men.

### Biomarkers of Variability

**FIGURE 11:**
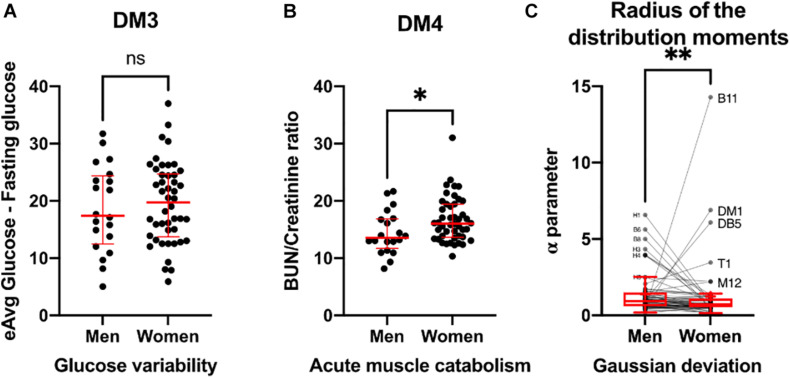
Biomarkers of variability. Glucose variability **(A)**, assessed by estimated average glucose minus fasting glucose is presented as individual values (black) with median ± 95% CI (red). Acute muscle catabolism **(B)**, and hydration state are summarized by BUN/creatinine ratio and presented as individual values (black) with median ± 95% CI (red). Deviation from Gaussian distribution **(C)** calculated as the radius of the distribution moments for each physiological variable is shown as individual values (black) and Tukey’s box-plots showing median and interquartile range. Physiological variables with visible separation from Gaussian behavior are labeled. Statistical significant difference is indicated by ^∗^ if *p* < 0.05, ^∗∗^ if *p* < 0.01 and ns for no difference (*p* > 0.05).

Thus far, we have observed differences in resilience and adaptability between men and women through time series variability and complex inferential networks. In addition, we sought to examine these differences through certain biomarkers ratios that are recognized as variability indicators, and by the differences in distribution moments of all the physiological variables in the population (Figure 11). For instance, glucose variability, assessed through the difference between estimated average glucose and fasting plasma glucose was similar in both healthy men and women (Figure 11A). In contrast, acute muscle catabolism, assessed through the ratio between blood urea nitrogen and serum creatinine was higher for healthy women than for healthy men (Figure 11B). Finally, for most physiological variables measured, deviation from normal distribution was lower in women than in men, except for body water % (B10), body mass index (DM1), total body water (DB5), and tympanic temperature (T1) (Figure 11C). Conversely, physiological parameters related to immune cells were non-normal in men (H0, H1, H3, and H4) approached more a normal distribution in women.

## Discussion

There is a close relationship between indices of sympathetic activity and vascular resistance in men as a function of age (Joyner et al., 2016). This relationship is absent in young women, but is noticeable in postmenopausal women. These sex and age differences are related to vascular resistance, and are the result of changes in the balance between the receptors responsible for arterial tone, i.e., vasoconstriction and vasodilation (Joyner et al., 2016). In young men, there is a relationship between neuromuscular sympathetic activity (NSA) and total peripheral resistance (TPR). However, this relationship is not associated with blood pressure, but is due to the fact that cardiac output is inversely proportional to NSA and TPR. In contrast, in young women, there is no relationship between NSA and TPR, due to the vasodilator mechanism of B-adrenergic receptors, which counteract the vasoconstriction of A-adrenergic receptors. Therefore, blood pressure is not related to NSA in young women (Joyner et al., 2016).

Sympathetic activity also affects the cardiovascular system; thus, it would produce different heart rate variability signals depending on age and sex. The cardiovascular system is one of the most regulated systems in the human body, and its study sets the tone for a great tradition of research and innovation. In the context of this characteristic integration of several systems, it has been established that significant differences occur between men and women in physiological regulation. The analysis of the time series of cardiac activity provides indicators of the functioning of the system and also of the effect of the parasympathetic and vagal modulation to which it may be subjected. The analysis of these fluctuations allows to know the state of these oscillators with respect to sympathetic and vagal modulation (Berntson et al., 1997). The analysis of the statistical moments provides indicators of the degree of rigidity or adaptability of the physiological system (Rivera et al., 2016, 2018).

For the case of Fantasia database, statistical moments shows that in young women RR is more rigid and less variable than for men, and that in old age the variability presented by men is lost, becoming a rigid system. With respect to Poincare’s plots, young men have the most elliptic distributions, related with low correlations, and old men the most linear ones indicating stronger correlations, rigid distributions. Women statistical parameters change less than the ones of men, suggesting that the heart rate variability is more “robust” and correlated.

In contrast, Shannon’s entropy for young subjects is in the same region for women and men, and it is statistically significant different between old women and men. The change of entropy between young and old women is small compare with the suffer by men. Considering Shannon’s entropy as a measure of the facility to transmit information on the time series, for a constant signal the value will be zero (that corresponds to a “death” subject), and the larger the value better information transmission will be in the system. Similar values of entropy for young subjects indicate a “good” homeostatic balance independent of sex, with aging this entropy have a slow decrease for women, but a stronger change in men, that goes for old men close to zero. This may be one of the many factors that made heart rate variability a vulnerability factor for death incidence greater in old men.

We have reported previously how node centrality measures show the role of physiological variables within the physiological networks, how these roles change with age (Barajas-Martínez et al., 2020), and how topological and algorithmic community detection reveals functional clusters (Barajas-Martínez et al., 2021). Through this methodology, we have constructed physiological networks to describe how the changes in the coupling between regulated variables and those regulatory systems that try to maintain homeostasis may differ between young healthy men and women. Here we interpret the lack of correlation as one variable not being dependent on another. Some correlations are suggestive of a vulnerability of the system to develop a pathologic state (even when all values are within normal parameters and disease onset has yet to happen), while others indicate a healthy physiological coordination. In the differential network we found sex-dependent differences in several nodes. We found that for men urea levels are correlated with BMI and related anthropometric indicators but not for women, being both equally young healthy subjects that have urea, creatinine, and eGFR within the normal range (Figures 6, 8). Likewise, for men creatinine was related to insulin and HOMA, but not for women. This is notable in the context of the urea/creatinine ratio, an indicative of acute muscle catabolism, being higher for women (Figure 11). This reinforces the hypothesis, as previously suggested in the literature, that men have a greater difficulty in maintaining homeostasis between food intake and uremic solute clearance for the same level of eGFR (Nitsch, 2014). Another difference we found between sexes was the correlation between blood pressure and hemoglobin in men (Figures 6, 8). This correlation has been found for both men and women in a large cohort of Dutch voluntary blood donors and was attributed to erythropoietin, renin-angiotensin-aldosterone system or the mechanic effects of blood viscosity (Atsma et al., 2012). It is possible that a larger sample is required to observe the effect on both sexes, or that the age difference with our sample is behind this discrepancy. Nonetheless, we notice that both erythropoietin responsiveness and renin-angiotensin-aldosterone have sex differences that may also play a role (Ifudu et al., 2001; Komukai et al., 2010). Only in men, plycometric measurement of skinfolds was correlated with HDL, estimated average glucose and HbA1c (Figures 6, 8), even when both sexes had similar glucose variability (Figure 11). Glucose and lipid metabolism are directly modulated by sexual hormones, resulting in different combinations of the clustering of metabolic syndrome factors (Regitz-Zagrosek et al., 2007; Kuk and Ardern, 2010). Finally, we observed different correlations of neutrophils in men and women. While neutrophil variables were associated with insulin, HOMA and plycometry in women, they were correlated with body composition measurements by bioimpedance in men. This may be due to differences in the adipose tissue patterns that depend on sex, with subcutaneous fat being more abundant in women and visceral fat in men (Lumish et al., 2020). These proinflammatory associations in neutrophils have been found for both sexes (Ibáñez et al., 2005; Xu et al., 2015). It is important to mention that the correlations discovered in this work are the result of a network that has been filtered using a *p*-value threshold. Other strategies, however, are possible. For example, a multiple comparison correction could be added to aid in the selection of significant correlations. Another strategy is to use a bootstrap value threshold to identify correlations that occur frequently and are thus more robust. Row bootstrap or pair bootstrap methods can be used for this purpose (Musciotto et al., 2018). We use a row bootstrap approach to determine whether the differences in topological properties between sexes are robust. This same procedure could be used to further filter the network’s correlations.

On the level of whole network analysis, we found that despite the apparent differences in connections between sexes, each physiological variable played similar roles in terms of influence (eigencentrality) and intermediation (betweenness). Additionally, algorithmic clustering was similar for both physiological networks (Table 4). Furthermore, the hierarchical arrangement of nodes shows a similar structure for both sexes (Figure 9). This suggests that global functions of the systems are largely the same. The most notable difference in topology results from the density and modularity of the network, as a result the physiological network of men has a greater small-world index (Figure 7). Consequently, the physiological network of men was more vulnerable to degree-directed attacks, and women’s physiological network was resilient to random failure (Figure 10). Lastly, deviation from normality was higher for physiological variables of men than those for women (Figure 11). All things considered, the physiological network of women indicates a more resilient homeostatic regulation that restrains regulated variables within strict ranges, and as a result, network properties such as small-world and modularity are decreased. Taking into account this assortment of approaches, we suggest important differences in the resilience and adaptability of physiological systems in men and women. In the context of the network representation of physiological systems, a network is resilient to attacks if it contains a large number of alternative paths that maintain the system’s connectedness and thus coordination. This increase in a system’s robustness comes at the expense of its component’s flexibility. An adaptable network has a higher modularity, allowing components to operate independently while retaining the small world topology that enables rapid communication within the network. This, however, exposes the network to targeted attacks. Figure 10 is a simulation of a directed attack with the purpose of highlighting differences between men and women in the structural response to attack in networks. One real-world example may be the different structural response to an infection with SARS-CoV2, where being male has been considered as a risk factor (Peckham et al., 2020), possibly due to a less resilient physiological network with respect to women.

It is important here to point out that the aim of our work is to characterize the function and properties of physiological systems in men and women. In this sense, the meaning of the words “adaptable,” “robust,” and “resilient” are not intended to describe individuals but the physiological systems. Additionally, when searching for sex differences, caution is advised in “big data” approaches to consider confounding factors that may be involved due to the risk of finding sex differences where none exist (type I error) (Rich-Edwards et al., 2018). To attenuate this risk, we performed a narrow-age cohort from a specific population, the participants were then filtered by health criteria presented on Tables 1, 2 to ensure a well-defined sample as much as possible. The database from which these healthy young subjects were selected has also an extensive questionnaire on socio-economic status and health risks. Finally, by using a network approach, conclusions are drawn in the context of the whole instead of relying in any single correlation in isolation. Despite the fact that bivariate similarity tests are easy to understand and represent, higher-order correlations cannot be taken into account by this analysis. As a result, in order to inspect these correlations, other methodologies would need to be added.

## Conclusion

We examined the differences between the sexes in the coordination of physiological systems by means of two methodologies: time series analysis of the specific variable of heart interbeat intervals and physiological networks constructed from point measurements of 62 different variables (50 variables directly measured related to anthropometry, bioimpedance, hematic biometry, blood chemistry, and 12 derived variables). We show consistency in the existence of sex differences both in the dynamics of heart rate as in the architecture of physiological networks. In youth, men have higher heart rate variability than women, as measured, e.g., with standard deviation or SD1 and SD2 of Poincaré plots, but with aging this variability is lost at a more rapid rate in men than in women. This effect is even more evident using Shannon entropy, which shows that entropy of heart interbeat intervals tends to be conserved in women but decreases drastically with aging in men. With respect to physiological networks, women have a higher connectedness, density and clustering coefficients, whereas men have higher modularity, eigencentrality and small-world index. Efficiency, node strength and measures that depend on the path structure of the network were similar for men and women. There were no differences in susceptibility to random failure between men and women, but the network of men was more vulnerable to directed attacks than the network of women. Both methodologies suggest that the physiological system of women has a greater number of connections, which translates into redundant regulation mechanisms and, on the other hand, has less variability with respect to that of men, that is, it is a more resilient system.

## Data Availability Statement

The original contributions presented in the study are included in the article/Supplementary Material, further inquiries can be directed to the corresponding author/s.

## Ethics Statement

The studies involving human participants were reviewed and approved by the Ethics Committee of the Facultad de Medicina of the Universidad Nacional Autónoma de México (UNAM) under project FM/DI/023/2014. The patients/participants provided their written informed consent to participate in this study.

## Author Contributions

AB-M, EI-C, and AR performed the network modeling and wrote the manuscript. CS did the conceptualization of the project for dataset of Project_42 and funding acquisition. AB-M and JL-R participated in data collection. AR, JL-R, RL, and JG did the data curation. AB-M, EI-C, JL-R, RL, JG, and AR performed the data analysis. AR, AB-M, EI-C, RF, JT-R, BE, OR-A, and AF participated in the complexity interpretation and analysis of the results. VM-G, GT-S, CA-S, BE, NT, AT, and MH participated in the medical interpretation. All authors contributed with the manuscript revision, read, and approved the final version of the manuscript.

## Conflict of Interest

The authors declare that the research was conducted in the absence of any commercial or financial relationships that could be construed as a potential conflict of interest.
