# Supplementary material for: Sex Differences in the Physiological Network of Healthy Young Subjects
Source: Front Physiol. 2021 May 11;12:678507. doi: 10.3389/fphys.2021.678507 (PMC8144508; doi:10.3389/fphys.2021.678507)
Supplement: Supplementary file 1 [file Table_1.PDF]

Supplementary

Sex differences in the physiological network of healthy young subjects

Table 1. Derived variables

Variable	Formula
Pulse pressure (PP)	$SBP_{(mmHg)} - DBP_{(mmHg)}$
Mean arterial pressure (MAP)	$\frac{[SBP_{(mmHg)} + 2 * DBP_{(mmHg)}]}{3}$
Body mass index (BMI)	$\frac{[weight_{(kg)}]}{[height_{(m)}]^2}$
Waist to hip ratio	$\frac{[waist_{(cm)}]}{[hip_{(cm)}]}$
Waist to height ratio	$\frac{[height_{(cm)}]}{[waist_{(cm)}]}$
Body fat	body fat $percentage_{(\%)}$ * $weight_{(kg)}$
Total body water	body $water_{(\%)}$ * $weight_{(kg)}$
HOMA-IR	$(glucose_{(mg/dL)}$* $insulin_{(\mu UI/mL)})/405$
Estimated glomerular filtration rate (wGFR)	In women, if $SCr_{(mg/dL)} \leq 0.7$ then<br>$eGFR= 144*[SCr_{(mg/dL)}/0.7]^{-0.329} * 0.993^{age_{(yr)}}$<br>In women, if $SCr_{(mg/dL)} > 0.7$ then<br>$eGFR= 144*[SCr_{(mg/dL)}/0.7]^{-1.209} * 0.993^{age_{(yr)}}$<br>In men, if $SCr_{(mg/dL)} \leq 0.9$ then<br>$eGFR= 141*[SCr_{(mg/dL)}/0.7]^{-0.411} * 0.993^{age_{(yr)}}$<br>In men, if $SCr_{(mg/dL)} > 0.9$ then<br>$eGFR= 141*[SCr_{(mg/dL)}/0.7]^{-1.209} * 0.993^{age_{(yr)}}$
Estimated average glucose	$[28.7 * HbA1c_{(\%)}] - 46.7$
Estimated average glucose – Fasting glucose	$eAG - glucose_{(mg/dL)}$
BUN to creatinine ratio	$\frac{BUN_{(mg/dL)}}{creatinine_{(mg/dL)}}$

Table 2. Differences in physiological variables between healthy men and women

	**Mean of Men**	**Mean of Women**	**Difference**	**SE of difference**	**Mann-Whitney U**	**Adjusted *p*-value**
Height	1.68	1.58	0.10	0.01	788	<0.000001
Total body water	39.9	30.2	9.7	1.0	298	<0.000001
Body fat percentage	19.5	28.9	-9.5	1.2	864	<0.000001
Waist to hip ratio	0.83	0.78	0.05	0.01	970	<0.000001
Weight	65.9	56.2	9.6	1.3	1092	<0.000001
Creatinine	0.99	0.76	0.23	0.03	18	<0.000001
Waist circumference	80.5	74.0	6.5	1.0	1143	<0.000001
Arm circumference	28.3	25.7	2.6	0.5	1360	0.0002
Body water	59.4	54.3	5.1	1.0	528	0.00004
Systolic blood pressure	111	105	7	1	1495	0.0002
Erythrocytes	5.6	4.8	0.8	0.1	12	0.002
Uric acid	5.69	4.47	1.21	0.24	68	0.0001
Hemoglobin	16.7	14.6	2.1	0.4	12	0.002
Body fat	12.9	16.5	-3.6	0.8	1649	0.01
Hematocrit	50	45	6	1	16	0.006
Mean arterial pressure	85	80	4	1	1657	0.009
Triceps plycometry	14	18	-4	1	1757	0.02

Table 3. Statistical parameters of the RR distributions from Fantasia Database

Group	μ (s)	SD (s)	CV	sk	K	median (s)	minimum (s)	maximum (s)	α
**young women**	0.9±0.1	0.08±0.03	0.10±0.03	-0.4±0.9	6±10	0.9	0.2	1.6	1.2±0.7
**young men**	1.1±0.1 *	0.11±0.04	0.05±0.02 *	-0.3±0.4	1±1 *	1.1	0.1	1.8	2.5±2.9 *
**old women**	1.0±0.1	0.05±0.01 *	0.08±0.02	-1.3±1.5	25±30 *	1.0	0.2	2.1	1.0±0.8
**old men**	1.1±0.2 *	0.08±0.05	0.05±0.02 *	-1.4±0.9 *	11±9	1.1	0.2	1.8	10±12 *

* indicates statistically significant difference with respect to young women group (*p*-value <0.05)

Table 4. Correlation and entropy parameters of the RR distributions from Fantasia database

Group	SD1 Poincare's ellipse	SD2 Poincare's ellipse	eccentricity Poincare's ellipse	Shannon's entropy
**young women**	33±11	97±28	0.93±0.04	1.5±0.1
**young men**	53±18 *	147±53 *	0.93±0.04	1.5±0.2
**old women**	28±8	63±15 *	0.86±0.1 *	1.4±0.2
**old men**	30±17	87±36	0.92±0.05	1.0±0.2 *

* indicates statistically significant difference with respect to young women group (*p*-value <0.05)
